# Modern instant messaging platform for postoperative follow-up of patients after total joint arthroplasty may reduce re-admission rate

**DOI:** 10.1186/s13018-019-1407-3

**Published:** 2019-12-27

**Authors:** Qing-Yuan Zheng, Lei Geng, Ming Ni, Jing-Yang Sun, Peng Ren, Quan-Bo Ji, Jun-Cheng Li, Guo-Qiang Zhang

**Affiliations:** Department of Orthopedics, Chinese PLA General Hospital, 28 Fuxing Road, Beijing, 100853 China

**Keywords:** Joint replacement, Total joint arthroplasty, Instant messaging

## Abstract

**Background:**

Follow-up after artificial joint replacement greatly helps achieve surgical outcomes. Mobile internet technology and mobile terminal equipment may increase the effectiveness of artificial joint replacement. However, only a few studies have evaluated the effectiveness of this technology. We aimed to analyze the reasons and outcomes of patients who used the instant messaging platform after undergoing artificial joint replacement.

**Methods:**

Among the 548 cases of arthroplasty (250 hips, 298 knees) performed between December 2015 and June 2018 in the Department of Joint Surgery of our institution; 358 (164 hip joints, 194 knee joints) participated in instant messaging platform consultation, whereas the remaining 190 (86 hip joints, 104 knee joints) participated in traditional telephone consultation, as a control group. Follow-up time was from December 2015 to August 2018 (follow-up period was 2–32 months). Data on age, sex, type of surgery, date of surgery, date of discharge, and length of hospital stay were collected from electronic medical records.

**Results:**

We analyzed the consultation contents of 358 patients who participated in instant messaging platform consultation. Counseling was mainly related to pain (13.6%), appointment review (12.4%), activity problems (10.5%), and incision problems (8.9%). Most problems were resolved through online guidance, with 8.4% of patients requiring only outpatient treatment and 2.5% of patients requiring rehospitalization. A total of 190 patients were followed up through traditional telephone consultation; 6.8% of patients required outpatient department treatment and 7.4% were eventually re-admitted.

**Conclusion:**

The instant messaging platform consultation service effectively informs patients of potential postoperative problems and helps resolve them. It allows early detection and management of postoperative adverse events, including problems related to medication, wound, and activity, thereby effectively reducing readmission rate.

## Introduction

In the past few decades, the demand for artificial joint replacement has increased dramatically, and the number of artificial joint replacement surgeries is expected to increase in the future in view of the fact that the patient population of artificial joint replacement is generally older [1]. With the aging of Chinese social population, the age of patient groups will generally increase. The establishment of a timely, effective, convenient, intuitive, and full-time follow-up model of artificial joint replacement is important. Fortunately, the development of modern instant messaging technology has made it possible to change the follow-up pattern after artificial joint replacement.

In addition, the average length of hospital stay after artificial joint replacement has decreased [2]. Modern artificial joint replacement technology allows surgical patients to be safely discharged within a short period of time after surgery [3, 4].This trend of shorter length of hospital stay emphasizes the importance of pre-discharge patient education. A large proportion of postoperative adverse events may be avoided and can be prevented by better patient education and enhanced postoperative counseling [5]. Telephone service is considered to reduce the medical cost of artificial joint replacement surgery, improve the efficiency of postoperative problem detection and resolution, and improve patient satisfaction. However, the actual effect of this service is still unclear. In our institution, we launched an instant messaging application consultation service for patients who have undergone artificial joint replacement to reduce unnecessary postoperative adverse events. Postoperative patients who received instant messaging platform counseling will receive verbal and written instructions on how to use this service.

Studies describing or evaluating similar instant messaging platform consulting services are rare [6]. A study confirmed that consultation phone service can reduced the number of unnecessary ED visits in patients after total joint arthroplasty [7]. Another study attempted to use automated text messaging to provide medical information to patients during the perioperative period, and they confirmed that patients with TJA who communicated during the perioperative period through automated text messaging had improved postoperative satisfaction [8]. The main purpose of this study was to introduce a new mode of postoperative follow-up-instant messaging platform, and analyze the reasons and outcomes of patients who used the instant messaging platform to guide our clinical work. Our second goal was to evaluate the effectiveness of the instant messaging platform consulting services by comparing the incidence of postoperative adverse events between patients who use the instant messaging platform and those who were followed up through traditional telephone consultations.

## Materials and methods

Our institution's research ethics board approved this study, and all patients participating in this project have chosen to be followed up and followed up according to their own wishes, and they have been informed of all relevant matters and obtained the consent of them. All joint arthroplasty operations in our institution are carried out according to standardized procedures. The patients are advised to move to the ground and walk with walker in the next day after surgery and are discharged at the discharge criteria, usually within 1 week after surgery. We collected data of patients (548 cases: 250 hip joints, 298 knee joints) who underwent hip and knee replacement at our institution from December 2015 to March 2018.These procedures were performed by two high-level surgeons specializing in hip and knee arthroplasty. The exclusion criteria were non-TJA (total joint arthroplasty) surgical cases, revision cases, those with primary infection, multiple site surgical cases, and those loss to follow-up. Of the 548 patients, 358 chose to follow up and consult through instant messaging, and the other 190 chose to register their phone numbers and conduct follow-up and consultation through telephone.

Before surgery, each patient received oral and written instructions on how to prepare for the surgery and how to recover. The attending doctor completes the preoperative form based on the patient’s condition and asks questions about previous surgery, existing medications, comorbidities, and illness at home. After the operation, patients were encouraged to be mobile as soon as possible, usually on the next day of surgery, and received standardized treatment every day during hospitalization. The patient was discharged at 3–5 days after surgery, when they met the discharge criteria (can stand up and walk short distance, has effective pain control, and can use the bathroom independently). At the time of discharge, the patient was given verbal and written instructions on how to use the instant messaging platform consulting service. Each patient also received postoperative guidelines including rehabilitation training and instructions for wound care, medication, and prevention of deep vein thrombosis and treatment.

We generally require patients who had undergone hip or knee surgery to use painkillers for 6 or 4 weeks, respectively. Similarly, patients undergoing hip and knee surgery receive rehabilitation guidance that varies from joint to joint.

In this study, we collected all patient data received through our instant messaging platform. The instant messaging platform was open 24 h, and data are received and registered by the surgical team’s doctor’s assistant. The information registered includes the name, the surgical site, the main reason for the contact, and the actions taken to resolve the patient’s problem. The main reasons for consultation were classified as medication, wound treatment, cost, insurance issues and other paperwork, swelling, sensation, (including pain), or activity. The options available for action taken to resolve the problem are as follows: the doctor’s assistant uses the application’s instant online guidance, consults the attending physician, asks for emergency treatment, asks for further hospitalization, or others. If the doctor’s assistant is unable to resolve the patient’s question, she will consult the attending doctor. The attending physician determines the patient’s next treatment. Patients who do not seek consult through the instant messaging platform will be followed up by the doctor’s assistant (6 weeks, 3 months, 6 months, 1 year, and yearly thereafter). The patient will be followed up or asked to return to the hospital for follow-up to determine the patient’s postoperative condition.

We used the electronic medical record system to collect information about age, sex, surgical site, type of surgery, date of surgery, date of discharge, and length of hospital stay. We encouraged patients to report back to us through the instant messaging platform as soon as possible, and asked patients to send videos, pictures, and voice messages instead of a single text to express their concerns.

We compared the incidence of emergency treatment and readmission events between the platform and telephone consultation groups to examine the effectiveness of the platform counseling and to control the incidence of serious adverse events.

## Statistical analysis

For statistical analyses, values of *p* < 0.05 were considered significant. Descriptive analyses of counseling content and methods of participants were conducted. Parametric data were assessed via the *t* test, whereas nonparametric categorical variables were analyzed with the chi-square test to make comparisons between the 2 groups (IBM SPSS Statistics, version 21.0.0.0).

## Result

**Fig. 1:**
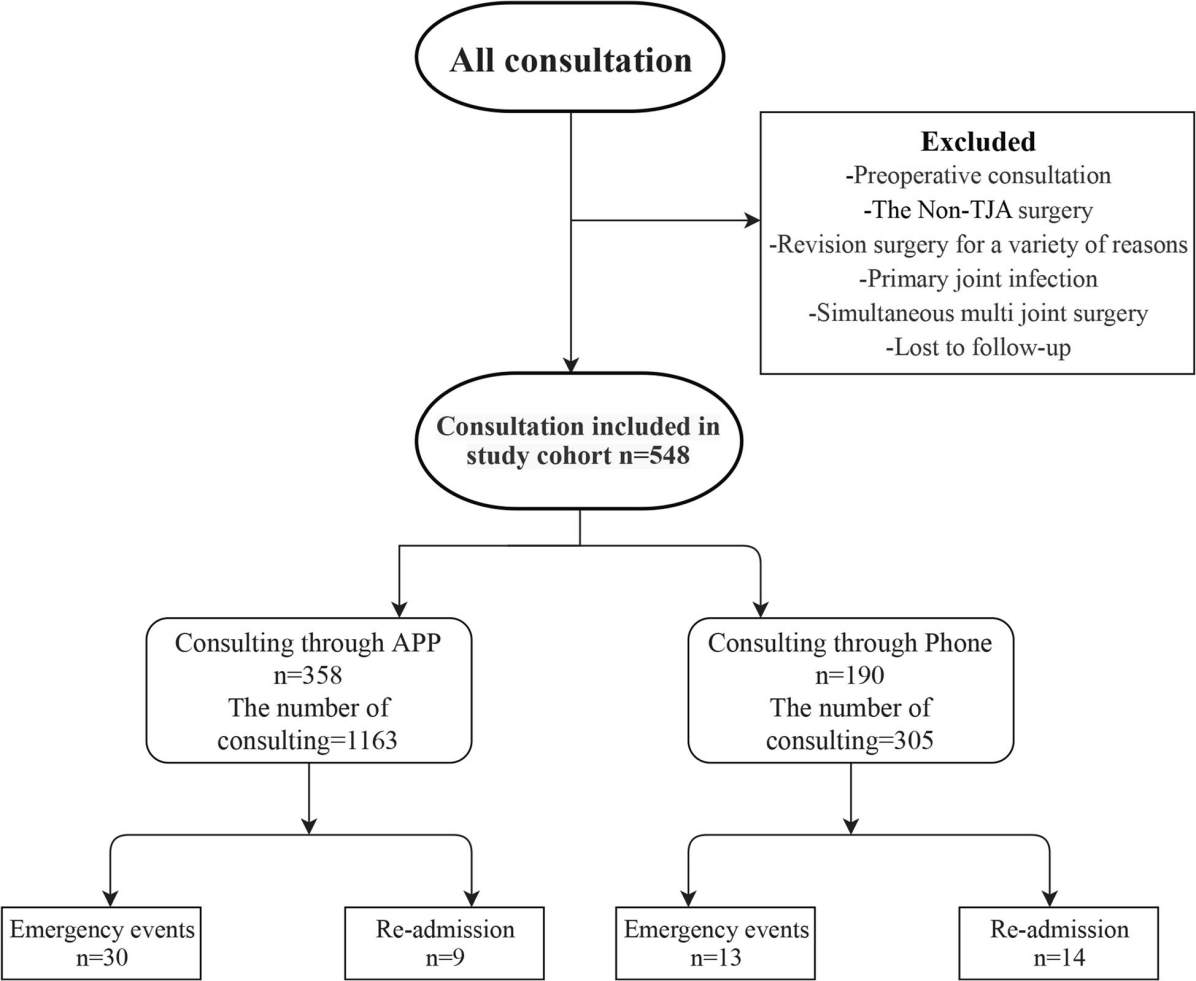
Flow chart of patients in the retrospective control study

During the study period, we included a total of 548 patients. According to the postoperative consultation follow-up model selected by the patients, 358 (consultation 1163 times) and 190 (consultation 305 times) patients comprised the platform and telephone consultation groups for analysis (Fig. 1). There were no significant differences in male/female ratio, age, or body mass index between the two groups.

In the platform consultation group, the average age of patients was 60.6 years (range 18–88 years); female patients accounted for 66.2% (237 patients), and 54.2% of the consultations were from patients who underwent knee surgery. In the telephone consultation group, the average age of patients was 62.6 years (range 23–84 years), female patients accounted for 53.2% (101 patients), and 54.7% of the consultations were from patients who underwent knee surgery. The average postoperative hospital stay was 5.7 days(range 3–16 days), and the average first contact time was 13.2 days (range 5–42 days) after the surgery. The longest postoperative contact time was 1.4 years after surgery.

**Fig. 2:**
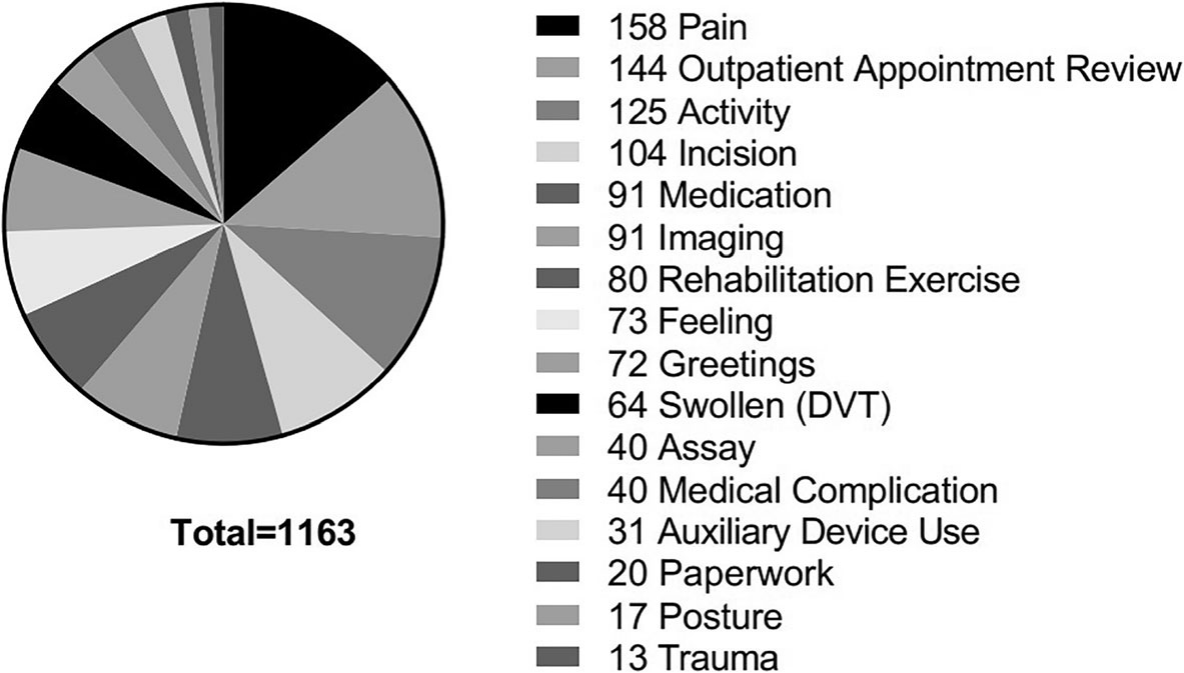
Postoperative instant messaging platform consultation common causes

A total of 1163 postoperative consultations were made through the instant messaging platform by 358 patients (Table 1). The three most common reasons for using telephone counseling services are pain- (13.3%), activity- (10.8%), and incision- (8.9%) related (Fig. 2). The time span for postoperative pain counseling was relatively long. The earliest pain-related counseling began on the third postoperative day. At 20 months postoperatively, patients still sought consult for pain-related problems. Among the incision-related telephone consultations, 71% were about dressing and suture removal, 21% were related to poor healing, and 8% were related to suspected surface infections. Most of the incision-related consultations were made within 2 weeks after surgery. Fewer patients sought consult for incision problems after 2 weeks of suture removal. The activity-related consultations mainly involved the flexion and extension function of the joints and the presence of joint stiffness and pain during the activity. The activity-related consultations were frequently made within the month after discharge. Most patients sought consult for activity-related problems within 3 months after surgery, and fewer consultations were made after 3 months postoperatively. Activity counseling included management or prevention of joint stiffness caused by poor recovery postoperatively.

**Table 1:** Consultation using APP reasons per category

Reason for consulting through APP	THA	TKA	Total	Percentage (%)
Pain	51	107	158	13.3
Outpatient appointment review	42	102	144	12.4
Activity	38	87	125	10.8
Incision	38	66	104	8.9
Medication	50	41	91	7.8
Imaging	30	61	91	7.8
Rehabilitation exercise	34	46	80	6.9
Feeling	22	51	73	6.3
Greetings	46	26	72	6.2
Swollen (DVT)	33	31	64	5.5
Assay	13	27	40	3.4
Medical complication	12	28	40	3.4
Auxiliary device	21	10	31	2.7
Paperwork	9	11	20	1.7
Posture	12	5	17	1.5
Trauma	8	5	13	1.1

*TKA*, total knee arthroplasty; *THA*, total hip arthroplasty; *DVT*, deep vein thrombosis. One patient consultation can be categorized as more than one call reason

The number of consultations for knee patients is 1.5 times more than that for hip patients. The most frequent reasons for knee patients to seek consult were pain- (15.2%), activity- (12.4%), and incision- (9.4%) related, whereas, for hip patients, these were pain- (11.1%), medication- (10.9%), and incision- (8.3%) related.

The platform consultation group comprised 358 cases, and the number of consultations made was 1163, with an average consultation time of 3.24 times. Meanwhile, the phone consultation group comprised 190 cases, and the number of consultations made was 305, with an average consultation time of 1.61 times. There was significant difference between the two groups (*p* < 0.05). (Table 2)

**Table 2:** Comparison of patient consultation times and outpatient postoperative review rate between instant messaging platform group and traditional telephone consultation group (patient consultation times comparison)

Approach to counseling	Total number of patients	Total number of consultations (telephone follow-ups)	Average	Standard deviation	*p* (T text)
APP	358	1163	3.24	2.52	-
Phone	190	305	1.61	0.86	-
Total	548	-	-	-	< 0.05

Regarding the incidence of emergency treatment, 30 patients (8.4%; 21 knees; 9 hips) in the platform consultation group and 13 patients in the telephone consultation group (6.8%; 6 knees; 7 hips) were required to visit the emergency department for various reasons. There was no significant difference between the rate of emergency events in the two groups (*p* = 0.524). (Table 3) Regarding the treatment, the events requiring emergency treatment mainly involved pain (7 cases), allergy (6 cases), and trauma (6 cases) (Table 4). Regarding the incidence of re-admission events ,9 (2.5%; 7 knees; 2 hips) and 14 (7.4%; 10 knees; 4 hips) patients in the platform and telephone consultation groups, respectively, were re-admitted to the hospital. There was significant difference between the rate of re-admission events in the two groups (*p* = 0.007). (Table 5) The following reasons led to the re-admission: incisions (6 cases), activities (4 cases), infections (3 cases), and so on (Table 4).

**Table 3:** Comparison of emergency incident rates and readmission rates between the two groups (the rate of emergency events in the two groups)

Approach to counseling	Total number of patients	Number of THA	Number of TKA	Number of emergency events	*p*
APP	358	164	194	30 (K21; H9)	
Phone	190	86	104	13 (K6; H7)	
Total	548	250	298	43	0.5240

*K* Patients undergoing TKA; *H*, Patients undergoing THA

**Table 4:** Postoperative emergency department treatment and stratification of reasons for re-admission

Reason for consultation	Total number of times	Emergency events	Re-admission	APP	Phone	*p*
Allergy	6	6	0	3	3	0.72
Incision	8	2	6	4	4	0.59
Pain	8	7	1	5	3	0.84
Activity	6	2	4	3	3	0.72
Infection	5	2	3	1	4	0.10
Thrombosis (DVT)	3	3	0	3	0	0.51
Hematoma	1	1	0	1	0	-
Trauma	7	6	1	5	2	0.95
Respiratory system	2	1	1	2	0	-
Digestive system	4	4	0	1	3	0.24
Nerve injury	6	4	2	5	1	0.62
Dislocation	4	2	2	3	1	0.91
Loose	1	0	1	0	1	-
Feeling	3	3	2	2	1	0.58
Heterotopic ossification	1	0	1	0	1	-
Eczema	1	1	0	1	0	-
Total	66	43	23	39	27	0.32

**Table 5:** Comparison of emergency incident rates and readmission rates between the two groups (the rate of re-admission in the two groups)

Approach to counseling	Total number of patients	Number of THA	Number of TKA	Number of re-admissions	*p*
APP	358	164	194	9 (K7; H2)	
Phone	190	86	104	14 (K10; H4)	
Total	548	250	298	23	0.0070

*K* Patients undergoing TKA; *H*, Patients undergoing THA

## Discussion

This study successfully identified the main reasons why patients seek consult through the instant messaging application after undergoing artificial joint replacement. The identification of common postoperative patient questions provides important information about how to further improve the clinical workflow of joint surgery. On the basis of our results, the areas that require further improvement and patient education are pain, activity, and incision-related issues, which are consistent with the results of some previous studies [7, 9]. However, our data slightly differed because patients who used the telephone consultation service frequently asked drug-related questions. Moreover, this may be due to the characteristics of telephone consultation; drug-related issues are more easily achieved through telephone consultation [10]. Our results reflect to some extent the actual problems of post-surgical patients who have undergone artificial joint replacement. These results can be used as a guide to lessen the confusion of patients after discharge and to improve pre-discharge patient education.

Our findings also show that the platform consultation services can effectively identify and resolve patient problems. Although the data showed that the number of emergency treatment events in the platform consultation group was slightly higher than that in the telephone consultation group, no significant difference was observed between the two groups (*p* = 0.524) (Table 3). However, in terms of controlling the readmission rate, the platform consulting group showed significantly better results than the telephone consultation group (*p* = 0.007) (Table 5). We believe that this result may be due to the fact that the platform consultation service is able to address the problems of postoperative patients more fully, comprehensively, and in a timely manner; hence, for patients requiring treatments in the emergency department are easily be identified. Although the incidence of emergency treatment events was higher in the platform consultation group, they had a lower re-admission than the telephone consultation group, because their postoperative problems were promptly intervened and processed.

In our study, the most frequent cause of postoperative emergency treatment and readmission in the patients who had undergone joint replacement surgery (Table 4) was pain, especially chronic pain, , which is consistent with the results reported in many literatures [11–14]. However, although pain, allergies, and other problems may lead to increased incidence of emergency treatment after the operation, they will not lead to re-admission; it is more likely that patients with incision-, activity-, and infection-related concerns are re-admitted [15–20]. We analyzed the causes of postoperative adverse events and found that there were no significant differences in the specific reasons between the two consultation follow-up processes, which may be related to the small sample size.

**Fig. 3:**
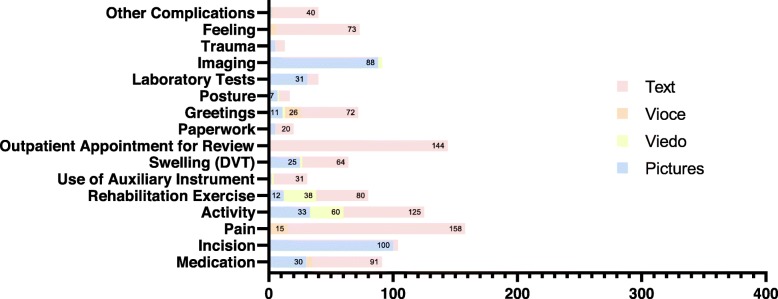
Different consultation reasons can be consulted on the instant messaging platform in different ways

Compared to the traditional telephone follow-up consultation model [10], the characteristics of modern instant messaging technology make it possible for us to fully and comprehensively understand the situation of postoperative patients. The interactive communication mode enables the postoperative patients to actively report their problems and status to the medical staff at the first try. However, for those patients without access to this service, they are passively and regularly followed up by medical institutions. The reliability and effectiveness of the patient’s active feedback are often higher with the modern instant messaging technology than with the traditional telephone follow-up consultation model [21]. Secondly, multimedia technologies such as use of pictures, videos, and voices are integrated into the same communication platform. Compared with the telephone follow-up consultation model (patients can only describe the problem by different languages), in the instant messaging technology, patients and their families can choose different ways to explain their status and problems (Fig. 3). For example, most patients choose to send pictures to show their incision problems, such as presence of swelling; those who perform active and rehabilitation exercises tend to send videos, and those with pain and other sensory problems, that are relatively subjective, use the voice function to directly explain to the medical staff; thus, the medical team has a certain degree of understanding of the patient’s feelings. In addition, when analyzing the average number of consultations between the telephone and platform consultation groups (3.24 vs 1.61 times, *p* < 0.05), our data showed significant difference, indicating that the patients utilized the instant messaging platform more frequently for consultation. Compared with the traditional consultation model, the use of smartphone-based instant messaging platforms is far more convenient than telephone and outpatient reviews, as it can accurately and directly identify patients’ problems and provide solutions in a short period of time. In our institution, our 24-h response rate has reached 90%, indicating that the platform consultation is also convenient for the medical staff, as they are more willing to communicate with the patient using the instant messaging platform.

Our results show that the platform consultation service is more easier to maintain long-term contact with the patient than traditional consultation. The postoperative outpatient postoperative review rate of patients in the platform consultation group is significantly different from that of the telephone consultation group (*p* < 0.05) (Table 6). The platform has established a relatively stable and long-term communication link with the medical staff. The traditional consultation model is relatively easy because of the patient’s replacement of contact methods and other reasons leading to loss of visits.

**Table 6:** Comparison of patient consultation times and outpatient postoperative review rate between instant messaging platform group and traditional telephone consultation group (outpatient postoperative review rate)

Approach to counseling	Total number of patients	Outpatient review	Outpatient visit rate	*p*
APP	358	214	0.597	-
Phone	190	64	0.336	-
Total	548	278	0.462	< 0.05

Our study findings will provide guidance to better improve the postoperative care of patients who had undergone prosthetic replacement, as the instant messaging application consultation service allows patients to receive immediate and satisfactory answers and guidance during subsequent consultations. Therefore, we believe that this service can make patients feel that they are valued, thereby promoting mutual trust between doctors and patients and improving patient satisfaction. In addition, compared to the traditional telephone consultation model, the rate of follow-up using the instant messaging platform was significantly reduced, thereby reducing medical costs and improving medical efficiency [6, 10, 22].

This study also has some limitations. First, patients who did not use the instant messaging application consultation service in this study had missing contact information and underwent a future outpatient review. The postoperative adverse events in these patients were not statistically significant. Second, many patients did not use instant messaging tools such as mobile phones because of old age and disability [22]. This affected our access to the information about the actual postoperative condition of patient to a certain extent. Therefore, it is particularly important to communicate with the family during the patient’s stay in the hospital. We suggest that these patients should try to avoid living alone, so that the family can assist them in using the application to timely give us feedback whenever problems arise. If an instant patient information feedback system based on family, community, and medical institutions can be established, the incidence of adverse events after artificial joint replacement will be further reduced, and the personal and social benefits of artificial joint replacement will be improved. Third, our study excluded cases of revision, primary infection, and multiple site joint replacement caused by various reasons. The common problems during postoperative consultation and follow-up in these patients are different from those of patients who underwent conventional joint replacement surgery [23, 24]. Finally, the number of cases is relatively small. We believe that the value and significance of this instant messaging platform consultation service need to be validate in further studies.

## Conclusion

Our data demonstrated that the instant messaging platform consultation service is more timely, effective, convenient, intuitive, and full-featured than the traditional consultation service. The consultation and follow-up mode based on the instant messaging platform has obvious advantages over the traditional model in timely discovering and dealing with postoperative problems, controlling the incidence of serious adverse events, effectively reducing the rate of readmission, and establishing a long-term stable relationship between doctors and patients. It can further reduce the medical cost of artificial joint replacement surgery and improve medical efficiency. It is a new postoperative patient consultation follow-up model worth further promotion.

## Data Availability

The datasets used and/or analyzed during the current study are available from the corresponding author on reasonable request.

## Abbreviations

TJA: Total joint arthroplasty
APP: Application
THA: Total hip arthroplasty
TKA: Total knee arthroplasty
DVT: Deep vein thrombosis
